# Triple Adaptation of the Mediterranean Diet: Design of A Meal Plan for Older People with Oropharyngeal Dysphagia Based on Home Cooking

**DOI:** 10.3390/nu11020425

**Published:** 2019-02-18

**Authors:** Alicia Costa, Silvia Carrión, Marc Puig-Pey, Fabiola Juárez, Pere Clavé

**Affiliations:** 1 Gastrointestinal Physiology Laboratory, CIBERehd CSdM-UAB, Hospital de Mataró, 08304 Barcelona, Spain; scarrion@csdm.cat; 2 Centro de Investigación Biomédica en Red de Enfermedades Hepáticas y Digestivas (CIBERehd), Instituto de Salud Carlos III, 08304 Barcelona, Spain; 3 Department of Dietetics and Nutrition, Hospital de Mataró, 08304 Barcelona, Spain; 4 Fundació Alicia, San Fruitós del Bages, 08272 Barcelona, Spain; marc@alicia.cat (M.P.-P.); fabiola@alicia.cat (F.J.); 5 Fundació de Recerca en Gastroenterologia, 08304 Barcelona, Spain

**Keywords:** oropharyngeal dysphagia, malnutrition, older, viscosity, texture, caloric, protein and organoleptic adaptation

## Abstract

Background: Oropharyngeal dysphagia (OD) and malnutrition are highly prevalent in older patients that are discharged from general hospitals (47% and 30%, respectively). Aims: To develop a nutritional plan for these patients involving a triple adaptation of their traditional diet: (a) rheological adaptation (texture and viscosity) for safe deglutition, (b) nutritional adaptation (water, calories, and proteins), and (c) organoleptic adaptation to improve compliance. Methods: Two fluid viscosities (250 and 800 mPa·s) were selected according to previous studies on optimal viscosities in older patients. The British Dietetic Association food texture classification based on common clinical practice selected two food textures (thick purée and fork-mashable. Two levels of calorie protein enrichment were selected according to previous studies using the Mini Nutritional Assessment (MNA^®^). Results: The daily caloric-protein and hydric needs were established at 1750 kcal, 70 g protein, and 1750 mL water in patients with MNA^®^ ≥ 17; and, 2037 kcal, 90 g protein, and 2000 mL water/day in malnourished patients. Sixteen weekly menus (296 recipes) were developed while using two textures, two levels of viscosity, two nutritional phenotypes (normal/at-risk vs. malnourished), and two seasons of the year (spring/summer-autumn/winter) based on Mediterranean cuisine. Conclusion: This concept paper demonstrates that traditional Mediterranean cooking can be adapted to meet the rheological, nutritional, and hydration needs of older patients with OD. The recipes that we have developed meet the needs of patients with varying degrees of OD and malnutrition are reproducible in patient’s homes and they could have a major impact on the clinical outcomes of these patients.

## 1. Introduction

Oropharyngeal dysphagia (OD) and malnutrition (MN) are highly prevalent, clinically relevant, and potentially treatable conditions among older hospitalized persons [1,2,3,4,5,6,7]. The World Health Organization in the International Statistical Classification of Diseases and Related Health Problems ICD-10 with specific codes (OD: R13 and MN:E40-E46) specifically classified both of the conditions [8]. The prevalence of OD varies between the phenotypes of older people: 60% in institutionalized patients [9], 47.5% in those that were admitted to a general hospital for an acute condition [10,11], and 27.2 in independently-living older people [12]. This prevalence increases in neurological patients, being over 80% in patients with dementia and from 29% to 64% in the post-stroke patients [13]. Two European scientific societies, the European Society of Swallowing disorders (ESSD) and the European Geriatric Medicine Society (EUGMS) recently recognized OD as a geriatric syndrome, due to its high prevalence and its relationship with many comorbidities and their poor outcomes, including malnutrition, dehydration, respiratory infections, aspiration pneumonia, functional disability, frailty, institutionalization, increased readmissions, and mortality [14].

MN is also a very prevalent condition among older persons that are admitted to general hospitals for acute diseases with a prevalence between 38.7%–80% [11]. OD has been shown to be an independent risk factor for MN in independent older people [12] and older patients that are admitted with an acute disease to a general hospital [11], and both MN and OD are associated with poor functional status, frail phenotype [11,12], and poor outcome with one-year mortality rates of 65.8% for patients with both conditions [11]. The pathophysiology of MN in older people with OD included starvation-related MN, chronic disease-related MN, and acute disease or injury-related MN [15], and studies have also found a significant association between sarcopenia and OD in older people [16,17,18]. The pathophysiology of OD in older people is also associated with slow neural response that delays pharyngeal reconfiguration [19] and with impaired pharyngeal sensitivity [20].

Current clinical management of patients with OD and MN that are discharged from acute hospitals is frequently poor and causes further complications [10,11,21,22]. In older people, OD is closely associated with community-acquired pneumonia (CAP), independent of functionality and comorbidities [4,21]. Recent data suggest that aspiration pneumonia due to OD and silent aspiration are important mechanisms for the pathogenesis of pneumonia in older people [23]. Our group has also found that nearly 5% of all hospital readmissions of older people, 27% of hospital readmissions for non-aspiration pneumonia, and 80% for aspiration pneumonia (AP) are attributable to OD, highlighting the relevance of this clinical condition in terms of health resource consumption [10].

We have recently developed a minimal-massive intervention (MMI) that aimed to treat a large number of older patients with dysphagia using simple interventions that are based on fluid and texture adaptation, nutritional supplementation, and oral hygiene [4]. As explained in previous publications, the philosophy of MMI is to maximize the number of patients that are treated because of the high prevalence of OD and MN in this population, by simple, cost-effective, and scientifically-proved measures [4]. The MMI is based on the early evaluation and treatment of the three main risk factors for OD complications: (1) dysphagia evaluation with a clinical tool (Volume Viscosity Swallow Test (V-VST)) and adaptation of fluids to avoid impaired safety of swallow; (2) nutritional evaluation and triple adaptation of traditional food (texture, nutritional content, and palatability) to improve patient nutritional status; and, (3) oral health and hygiene evaluation and treatment to avoid respiratory pathogen colonization of the oral cavity [24,25]. The results of our first proof of concept study with MMI on discharged older patients with OD was very positive as the MMI improved nutritional status and functionality and reduced hospital readmissions, respiratory infections, and mortality [4].

The aim of this study was to develop a nutritional plan for older people with OD, which was reproducible at home, and with a triple adaptation of home cooking: (a) rheological adaptation, for fluids—viscosity—and solids—texture—for safe swallowing, (b) nutritional adaptation to meet the nutritional requirements of older people with OD, and (c) organoleptic adaptation, to optimize the taste, smell, presentation, and palatability of the dishes. The triple adaptation will systematize the outpatient treatment of older patients with OD that were discharged from acute hospitals to avoid aspirations and improve their nutritional status and quality of life and reduce respiratory complications and hospital readmissions.

## 2. Materials and Methods

To develop a nutritional plan for older patients with OD that safely meets the main nutritional and hydration requirements and is palatable, we used two caloric/protein enriched diets for normal/at risk nutritional status vs. malnourished patients based on a previous study [2] using the Mini Nutritional Assessment (MNA^®^); two levels of viscosity for a safe fluid adaptation based on previous studies [6,26] using the V-VST and videofluoroscopy [27] and two levels of food texture—thick puree and fork mashable—for solid foods from the BDA food texture classification [28] and as commonly prescribed in our clinical practice. Patients will be designated the correct diet according to their MNA, V-VST, and chewing ability results. The following adaptations will be made to food and fluids.

### 2.1. Rheological Adaptation

#### 2.1.1. Adaptation of Fluids: Total Water Requirements, Bolus Volume, and Viscosity

(a) Selection of the total water requirements. The amount of water/fluid per patient is determined according to their energy intake (mL/kcal) [29], as 1 mL/kcal [29,30,31].

(b) Selection of the bolus volume and viscosity. Thin liquids (water) and two levels of thickened fluids, 250 mPa·s and 800 mPa·s, were selected according to our previous studies assessing the therapeutic effect of fluid thickening in up to seven different levels of viscosity for this study [6,26]. Patients with OD will be prescribed the appropriate viscosity level according to the results of the V-VST [27], a validated bedside clinical test with optimal psychometric properties [32] that can select the optimal bolus volume (5, 10, or 20 mL) and viscosity to provide the safest and most efficacious swallow [33].

#### 2.1.2. Adaptation of Solid Foods: Food Texture

We selected two textures from the National Descriptors for Texture Modification in Adults that were previously described by the British Dietetic Association [28,34]: Texture C (thick purée) and Texture E (fork mashable), as they are the most used in our care settings and centres. Texture C does not contain lumps or hard pieces, like shells, bones, or fibrous pieces of the food itself. To achieve this texture, it is usually necessary to sift and strain mashed food to achieve a suitable homogeneous texture. The dishes are not usually garnished; rather they are usually a “single piece”. In Texture E, only a fork is necessary to turn it into "puree texture" with soft, tender, and moist characteristics. It should be served with a thick or creamy, but always homogeneous, sauce, i.e., without double textures or separate liquids. It should not contain seeds or skins in the food, or be surrounded by an edible skin of different texture to the rest of the food. Foods of these textures do not take their original forms and they should not contain hard pieces of food. They cannot include juicy foods that release liquid in the mouth [28].

Cooking techniques that are used in the Texture E dishes, such as boiling, steaming (papillote), bain marie, and stewing provide the optimal textures. Cooking methods that are less used for Texture E are griddled, baked, sautéed, or fried, as they dry the food or form a crust, with additional risk when swallowing. For Texture C, the usual cooking techniques of the dish are prioritized (stewed, fried, grilled), as it will be ground afterwards. The maximum variety of cooking methods was incorporated to avoid the frequent overuse of boiling. Patients with OD will be prescribed the appropriate texture according to the clinical chewing evaluation.

### 2.2. Nutritional Adaptation: Calories and Proteins, and Daily Distribution. Use of Oral Nutritional Supplements (ONS)

#### 2.2.1. Amount of Calories and Proteins

We have based our recommendations on previous data on nutritional needs that were obtained by our group in a representative sample of hospitalized older patients with OD while using anthropometric measurements, biochemical parameters, body composition (bioimpedance), and energy demand of this population [2]. Using these results, we created two diet categories according to the patients’ nutritional status: a category for patients with MNA^®^ ≥ 17 (nourished were grouped together with at risk of malnutrition due to the similarity in their usual weight (average 70.1 kg)), vs a category for MN patients (average weight 58.2 kg). Dietary energy requirements (DER) were calculated from the factorial estimates of the physical activity level. They were converted into energy units (calories) by multiplying the physical activity level by the basal metabolic rate, as obtained from basal metabolism data measured by bioimpendance. Dietary energy requirements of the two groups of patients (MNA^®^ ≥ 17 and MNA^®^ < 17) were calculated according to basal metabolism that was obtained by bioimpedance and the physical activity level. Following the recommendations of protein requirements by the PROT-AGE study on chronic disease, we established the requirements as 1.2 g/kg/day for older patients with MNA^®^ ≥ 17 and 1.5 g/kg/day for those with MNA^®^ < 17 [35]. To meet these needs, high energy density and protein nutrients are necessary, and ONS should be used when normal intake is insufficient [36].

#### 2.2.2. Energy Distribution (ED)

The ED of the daily menu for older patients with OD and MNA^®^ ≥ 17 was (a) 25–30% (438–525 kcal) of the energy at breakfast, (b) 35–40% (612–700 kcal) at lunch, (c) 25% (437 kcal) at dinner, and (d) 5% (88–263 kcal) in snacks [37]. To achieve this nutritional contribution, we had to prepare dishes with high nutrient density. The contributions of carbohydrates, lipids, and fibre were also taken into account with a distribution of 50–55% carbohydrates (350–385 g/day), 30–35% lipids (472–551 g/day), and a fibre content of 20 g [38]. The recommended daily portions were included, as well as the frequency of recommended daily and weekly consumption of farinaceous, fruit and vegetables, protein foods, dairy products, and fat, mainly olive oil [39].

#### 2.2.3. Oral Nutritional Supplements (ONS)

Our nutritional intervention is based on the use of menus that are made with traditional foods, including enriched diets for which we used a homemade snack. A “natural” supplement of 430 kcal and 20 g of protein has been added to the diet of the malnourished patient group (MNA^®^ < 17). However, we included ONS in the following situations, according to the latest ESPEN guideline on clinical nutrition and hydration in geriatrics: (a) very inadequate intake (<50%), or (b) severe loss of weight (LW) (>5% LW/month), or (c) insufficient intake (50–80% under requirement) of prescribed diets or significant loss of weight (LW) (5% LW/month) in patients with MNA^®^ < 17. This was based on the literature that indicates that weight loss during acute illness and hospitalization can be prevented by the provision of food of high energy and protein density, combined with snacks, and by the use of ONS when normal intake is insufficient [40,41,42]. We developed an algorithm to easily select the correct diets according to patients’ nutritional status, appetite, and oral intake (Tables S1 and S2).

### 2.3. Organoleptic Adaptation: Mediterranean Diet

We used the same typical foods of the Mediterranean diet [43,44] to create dishes the patients would be familiar with, while taking care to preserve their smell and taste and then present them in an attractive way, while at the same time adapting them to textures safe for the patients to eat. The aim was to improve compliance, return the pleasure of eating to these patients, and thus improve their quality of life.

The sensory validation was carried out by a multidisciplinary team that consisted of two cooks, a dietitian and a food technologist, from Alicia Foundation (Alice, Alimentation and Science) [45]. Each of the dishes went through a validation process that consisted of (1) a texture validation (based on the BDA), (2) an organoleptic validation where the team tasted the modified dish and validated that it had the same taste as the original dish, and (3) a second verification of texture, ensuring that it met the requirements of texture in the mouth (cohesiveness, hardness, elasticity, homogeneity, …). Each dish was valued and commented with the rest of the team. If the entire team did not validate the plate, it was modified, and the validation was then repeated. No validations with patients were performed.

### 2.4. Education Materials for Patients and Relatives. Video-Recipes and Cooking Shows

In order to facilitate the use of the recipes, we collaborated with “l’Escola Superior d’Hosteleria de Barcelona” (ESHOB), a culinary school, to develop video-recipes in plain language to show how to prepare each recipe in less than three minutes. Cooking workshops at Mataró hospital and in public markets were organized for relatives and caregivers to see how to produce a balanced and adequate calorie and protein menu [2,35].

### 2.5. Putting It All Together: Recipes and Menus

The process to create the recipes and menus of these four diets (risk of MNA, MNA, spring/summer, autumn/winter) followed five steps:

Step 1: Design of the structure of meal plan. First, we distributed the caloric intake in 4–5 meals a day, ensuring that we covered the recommended energy distribution percentages [37]. Second, ten food groups (vegetable, pasta, rice, potato, meat, fish, legumes, eggs, fruit, and dairy) were distributed in four meals that were based on the recommended frequency of weekly consumption from food guidelines [37]. We maximized food variety to enhance the pleasure of eating, and also increase food and energy intake and, in the short term, alter energy balance [46].

Step 2: Basic recipe design. We proceeded to design triply-adapted recipes with the collaboration of Alicia Foundation [45]. Three groups of recipes were designed: (a) Simple recipes with minimal adaptation where the easy preparation for organoleptic appeal was prioritized. Basically, they consist of recipes of fifth range products or fresh or slightly modified natural products. The aim of these recipes was to facilitate compliance of diet when culinary skills were limited; (b) Elementary recipes consisting of bases of food (meat, fish, vegetables, pasta, etc.) combined with sauces. The purpose was to facilitate the daily elaboration of the recipes by those with an average culinary level; and, (c) Innovative recipes, traditional dishes that are usually avoided in this population because of the risk of choking. Textural validation was carried out with the criteria of the BDA using a fork and a plastic spoon. The nutritional validation was carried out through the diet program (PCN-Cesnid 1.0) that was based on the Food Composition Tables of CESNID [47], the Food Composition Tables of the USDA [48], and the nutritional composition data on the labels of the food packaging. All of the recipes were reviewed one by one to ensure textural and organoleptic compliance and then refined to achieve the maximum possible nutritional density without losing organoleptic quality.

Step 3: Design of the diet plan. Finally, we proceeded to the creation of biweekly menus. We started by constructing the main meals of the menu (lunch and dinner), then breakfast, and then added deserts and snacks in order to cover: (a) daily caloric-protein requirements, (b) daily food portions that are recommended for older patients, (c) frequency of weekly consumption of the food groups (with colour coding to easily identify the different food groups), and (d) seasonality. We verified that all of the menus were healthy and specific for the older population, and so they were adjusted to healthy eating guidelines (distribution in 4–5 meals, weekly variety, well-balanced, adequate amounts of different food groups) [37,49], and met the characteristics of the Mediterranean diet (rich in plant foods, use of olive oil, use of seasonal foods, and use of simple recipes [50]. In terms of the frequency of food consumption, those food groups with the highest caloric and/or protein intake had to be prioritized over groups with lower caloric intake, such as vegetables. Weekly variety had to be suited to the satiety and habits of this population. More than 60% of the proteins in the menus had to be of high biological value (eggs, dairy products and derivatives, meats and meat products, and fish) to increase protein synthesis [51].

Step 4: Design of menus for two patient phenotypes. We designed sixteen menus, the first eight being for patients MNA^®^ ≥ 17, and then eight for malnourished patients, introducing “home-made” nutritional supplements (with approximately 415 kcal and 18 g of protein) and readjusting the daily and weekly foods. Homemade nutritional supplements were elaborated with dairy (quark or equivalent), fresh and dry fruit (apple and raisin or equivalent), nuts (almond or equivalent), farinaceous (sliced bread or equivalent), and extra ingredients (i.e., ground cinnamon). Oral nutritional supplementation (ONS) should be included in these menus, as previously described.

Step 5: Prescription of triple adaptation diets. All patients over 70 years of age that were admitted to an acute care hospital will be given the three clinical procedures: MNA-sf screen, V-VST clinical evaluation, and the clinical chewing evaluation.

Depending on the results of these three procedures, patients will be prescribed the appropriate diets according to nutritional status (caloric and protein adaptation), severity of dysphagia (fluid adaptation), and chewing capacity (level of texture modified food). (Figure S1). Patients with signs of aspiration not responding to compensatory strategies will be referred for a Videofluoroscopy (VFS) or Fiberoptic Endoscopic Evaluation of Swallowing (FEES).

All of the patients over 70 years of age should undergo these procedures on a regular basis. (1) The patients are systematically screened for nutritional status using the MNA-short form and, according to the result, will be prescribed a standard or an enriched diet. Patients who, in addition to being malnourished, have low intake and poor appetite and should take a fortified diet (enriched diet with an Oral Nutritional Supplement); (2) Patients are systematically evaluated for dysphagia using the V-VST and those with alterations in swallowing safety will be prescribed the optimal viscosity (250 mPa·s or 800 mPa·s) for fluids according to the degree of dysphagia severity that was detected by the test; and, (3) Patients with alterations in swallowing will undergo clinical evaluation of their chewing capacity, and in the case of dysfunction, will be prescribed Texture C or Texture E for solid food, accordingly. Patients with severe signs of aspiration will be referred for instrumental assessment with VFS or FEES when more information is needed to clarify the pathophysiology of swallow impairment and the mechanisms of aspirations. In the case of older patients with mild aspirations who can be managed by compensatory treatment, these diagnostic tools will not need be systematically applied.

## 3. Results

Following our triple adaptation (texture, nutritional value, palatability), we created 296 recipes, consisting of: 15 basic dishes combined with 27 sauces, 21 complete recipes, eight recipes with products from the fifth range, six recipes for breakfast, nine desserts, seven high caloric and protein smoothies, and three versatile recipes for Texture C, and 15 bases combined with 27 sauces, 21 complete recipes, six recipes for breakfast, and 10 desserts for Texture E, with all of them being based on the Mediterranean Diet. Figures S2–S5 show examples of recipes with triple adaptation.

Using the recipes, we developed four types of texture-modified diets: (1) Thick purée diet (Texture C) for patients with MNA^®^ ≥ 17, (2) Thick purée diet for patients with MNA^®^ < 17 (malnourished), (3) Fork Mashable diet (Texture E) for patients with MNA^®^ ≥ 17, and (4) Fork Mashable diet for patients with MNA^®^ < 17 (malnourished), divided between spring/summer and autumn/winter seasonal dishes. We produced sixteen weekly menus (Tables S3–S6) that were developed with two textures (thick purée (C) and fork-mashable (E)), two nutritional phenotypes of patients (normal and at-risk vs. malnourished according to the MNA^®^), and two seasons of the year (spring/summer-autumn/winter). These are the results of the four types of adaptations that are outlined in the Material and Methods section.

### 3.1. Adaptation of Fluids for Hydration: Total Water Requirements, Bolus Volume and Viscosity 

The total water requirements were established at 1750 mL water/day in older patients with OD and normal nutritional status or at risk of malnutrition and 2000 mL water/day in malnourished patients [2]. To adapt the viscosity of fluids, the chosen thickening agent for this study was Nutilis Clear^®^ that was composed by xanthan gum, which is resistant to salivary α-amylase [26,52], and the levels of viscosity that were selected for this plan were 250 and 800 mPa·s [6,26], prescribed according to the results of the V-VST in previous studies.

### 3.2. Adaptation of Solids: Texture Modified Foods 

Texture modification was developed in each group of diets to adapt the food textures to BDA guidelines [28]. Breakfast consisting of bread, cereal, cookies, or muffins was blended to be suitable for both textures (C and E). Recipes that are based on pasta or rice were blended in Texture C, but were allowed to be extra-cooked and lubricated with sauce in Texture E. However, potato recipes had to be mashed with a fork to improve the texture and a gravy or oil added to facilitate swallowing. In general, legumes were blended and strained to avoid skins, although a special red lentil (without skin) was suitable for Texture E.

Different viscosities can be obtained by blending food, but we produced purée viscosity to achieve a high nutritional content. Texture E vegetables were prepared without seed or skins, and we verified that they were tender before serving. Vegetables with skins, such as spinach, lettuce, or peas, were included in Texture E menus, but they had to be mashed first, like all vegetables for Texture C. Meat products were puréed and gravy was added for Texture C, or tender meats were chopped with gravy for texture E. The main meats that were used in the menus were turkey, chicken, and pork (less frequently beef) with a sauce, “botifarra”—catalan sausage—and hamburger. Fish was pureed for Texture C and then cooked whole with a sauce for texture E. Omelettes and croquettes were suitable for texture E so long as the surface was not browned. Commercially available yoghourts without lumps (fruits or cereals) and skyr or Greek yoghourts, crème fresh, or cottage cheese or “ricotta” were included in both diets (C and E), with the exception of natural yoghurt, which was omitted for patients that were limited to nectar viscosity.

Other desserts, such as custard or crème caramel (without burnt sugar), were used in menus due to their high acceptance among the older population. Finally, fruit was blended for both of the textures to avoid liquids being released in the mouth, and banana or baked fruit was included in texture E.

### 3.3. Adaptation of Alimentary Fluids

Alimentary fluids are preparations (mainly milk, juices, milk coffee, soups, and vegetable creams, like gazpacho and vichyssoise), which are then thickened with powdered cereals, semolina, bread or mashed potatoes, and/or thickeners. The viscosities that were chosen for the majority of these alimentary fluids, 250 mPa·s and 800 mPa·s, were those found to be optimum in older patients in previous studies [6], and the use of foods to thicken was prioritized to increase the caloric and protein intake (Table S7). Recipes for soups and purees were elaborated as thick purees (Texture C) in order to obtain the maximum nutritional contribution and avoid protein and caloric dilution.

### 3.4. Nutritional Adaptation: Calories and Proteins, and Daily Distribution. Use of Oral Nutritional Supplements (ONS)

We calculated a DER of 1750 kcal/day (25 kcal/kg/day) for the group of patients with MNA^®^ ≥ 17. This value was compared with the results that were obtained with the three theoretical formulas: (a) Harris–Benedict (HB) equation, (b) the Mifflin–St Jeor equation, and (c) the simplified formula of 25 kcal/kg, with similar results that confirmed our previous results. Patients with MNA^®^ < 17 needed an increase in calories due to their low weight, so we developed an intensive nutritional intervention of 35 kcal/kg/day [53,54,55] that gave an increment of 519 kcal, obtaining a corrected DER of 2.037 kcal. To summarize, based on our previous studies and the state of the art, we calculated the optimal caloric and the protein needs for older patients with OD and MNA^®^ ≥ 17 were 1750 kcal (25 kcal/kg/day) and 70 g of protein/day. In malnourished older patients (MNA^®^ < 17), caloric supplementation of 519 kcal/day and 20 g protein/day were necessary and then administered in fortified diets or nutritional supplements. We calculated the average contribution of the sixteen menus and compared the nutritional contribution of the menus with the recommended daily intake for older patients with OD. Standard menus provided 1750.37 ± 10% kcal/day and 72.64 ± 0.1 g of protein/day for Texture C menus and 1752.58 ± 10% kcal/day and 71.61 ± 0.1 g of protein/day for the texture E menus.

The high caloric and protein menus, including homemade oral nutritional supplementation (ONS) of 415 kcal and 18.3 g of protein (Figure S6), provided 2165.37 ± 10% kcal/day and 90.94 ± 0.1 g of protein/day for Texture C menus and 2167.58 ± 10% kcal/day and 89.91 ± 0.1 g of protein/day for the Texture E menus. We developed smoothies of quark, apple, ground cinnamon, almonds, raisins, and sliced bread. (Figure S6) In those patients who, in addition to severe malnutrition, had severe anorexia or very low intake (less than 50%), we included more intensive supplementation, with the use of a pharmaceutical product. In our diets, we have designed the intervention with Nutilis Complete^®^ (306 kcal, 12 g of protein, and 4 g of fibre) to be included according to the algorithm that is described in Tables S1 and S2.

Briefly, the procedure to select the correct diet for the patients will follow the indications of the algorithm. All of the patients will be evaluated at each visit and the nutritional recommendations will be based on these evaluations.

(a) During the screening visit nutritional status will be assessed with the MNA^®^-sf, obtaining three nutritional levels. Well-nourished patients (MNA^®^-sf 14–12) and those at risk of malnutrition (MNA^®^-sf 8–11) will follow the 1750 kcal 70 g protein menu. Malnourished patients (MNA^®^-sf < 8) will follow the 2037 kcal 90 g protein menu (enriched menu). For those patients presenting MNA^®^-sf < 8 and severe anorexia, or very low intake, will have a Nutilis Complete^®^ supplementation according to their intake (≥1 unit/day) (Table S1).

(b) In the follow-up visits, oral intake will be controlled with a graphical oral intake registry and then evaluated by the study nutritionist. Nutritional recommendation will be done according to the oral intake and to MNA^®^. Oral intake will be divided into three categories (good intake: 100–75%; insufficient intake: 75–50% or 5% weight loss/month; and, very low intake: <50% or >5% weight loss/month), as well as the MNA^®^ (well-nourished: >24; at risk of malnutrition: 17–24; malnourished: <17). Patients with an MNA between 24–17 with a good oral intake will follow the 1750 kcal 70 g protein menu, but those with an insufficient oral intake will follow the 2037 kcal 90 g protein menu (enriched menu). Those with a very low oral intake will follow the 2037 kcal 90 g protein menu (enriched menu) + 2 or 3 ONS (Nutilis Complete^®^) per day according to their intake. Patients with an MNA^®^ < 17 with a good oral intake will follow the 2037 kcal 90 g protein menu (enriched menu), but those with an insufficient oral intake will follow the 2037 kcal 90 g protein menu (enriched menu) + 1 or 2 ONS (Nutilis Complete^®^) per day according to their intake. Finally, those with a very low oral intake will follow the 2037 kcal 90 g protein menu (enriched menu) and ≥3 ONS (Nutilis Complete^®^) per day, according to their intake (Table S2).

### 3.5. Organoleptic Adaptation

Taste, smell, and palatability are the characteristics prioritized in simple basic recipes. However, innovative recipes also add colour and presentation. Simple recipes, such as “Modified commercial cream” or “Toast, butter and jam” focus on taste, and elementary recipes, such as “Chicken with mushroom sauce, garlic and parsley” or “Pasta with tomato and anchovies” focus on presentation. Elementary minced recipes, which are made of bases and sauces, are presented with the base on the bottom and the sauce on top in an attractive shape like a sun or tears. However, innovative recipes, such as green salad (Figure S7) or croquettes, imitate the traditional dishes and often use moulds or attractive plates and glasses their own skins like half an orange (ex. Fresh orange) (Figure S8).

### 3.6. Education Materials

Fifty video-recipes that were made by the Superior School of Hotel industry of Barcelona (ESHOB) can be seen at the following link: Video Recipes. We also developed cooking workshops, which were highly appreciated by patients, relatives, and caregivers. A video of one of the workshops can be seen here: Cooking workshops.

So far, we have carried out eight cooking workshops; three were dynamic (where patients were actively cooking) and five were demonstrations (where patients watched how the recipes were prepared). Main interest centred on how to prepare raw foods (for example, a salad), protein foods (mainly meat), and how to enrich normal food, such as milk coffee and soups. Workshop attendance has grown exponentially with 43 attendants at the last workshop. At the beginning, the audience was primarily women (wives and/or carers of patients), but the last workshop included health professionals and cooks. Feedback surveys of the workshops showed the highest level of satisfaction (10/10).

### 3.7. Menus and Recipes

Tables S3–S6 show an example of each of groups of menus, according to texture, season, and nutritional status. Menus include simple preparations (commercial vegetable creams, adapted soups, or ham and cheese sandwich—“bikini”), as well as more elaborate dishes (Catalan roast chicken, white beans with cod—“empedrat”—or rice with fish–“paella”), and modern preparations (salmon with saffron, chicken with soy and honey, or green salad), in order to promote active home cooking. We also included traditional recipes (potato omelette, chickpeas with spinach, noodles with fish—“fideuà”—or bread with “wine and sugar”). Figures S2–S5 show examples of recipes of both textures. The complete description of the process of preparation of each recipe will be found online at Video Recipes.

To prescribe the most appropriate initial diet for each older patient, two types of evaluation are recommended. First, an evaluation must be done through a validated nutritional screening tool, such as the MNA-sf. Those patients who present a good nutritional status or risk of malnutrition (MNA-sf = 8–14) will be prescribed the standard diet (1750 kcal and 70 g protein); and, malnourished patients (MNA-sf < 8) will be prescribed the enriched diet (2037 kcal and 90 g of protein) The second evaluation uses the patient’s records of intake in order to assess the patient’s current appetite and intake. Those patients with severe anorexia or an insufficient (75–50%) or very low intake (<50%) should receive an Oral Nutritional Supplement in addition to the enriched diet, as indicated in Tables S1 and S2. In the follow-up visits, weight must be monitored in addition to the previously mentioned parameters; a weight loss ≥5% per month is an indication for prescribing more units of oral nutritional supplements. (Table S2).

## 4. Discussion

This study shows that it is possible to adapt the traditional Mediterranean diet and develop recipes that can be produced in the home and that cover the rheologic (viscosity and texture) and nutritional needs of older patients with OD and MN. Sixteen weekly menus (296 recipes) were developed that were based on Mediterranean cuisine and included two textures (thick purée and fork-mashable), three levels of viscosity for fluids (thin liquids, 250 and 800 mPa·s), two nutritional phenotypes (normal and at-risk vs. malnourished), and two seasons of the year (spring/summer-autumn/winter). The daily caloric-protein and hydric needs were established at 1750 kcal, 70 g protein, and 1750 mL water/day in patients with MNA^®^ > 17; and, 2037 kcal, 90 g protein, and 2000 mL water/day in malnourished patients. Fluid viscosity for hydration and alimentary fluids was prescribed according to previous studies using the volume-viscosity swallow test. Food texture (thick purée and fork-mashable) was selected from the previously described Food Texture Descriptors of the BDA. The calories and proteins were prescribed according to previous studies using the Mini Nutritional Assessment (MNA^®^). Recipes for these diets have been designed to be produced at patients’ homes and we believe that they might have a major impact on the clinical outcomes of these elderly patients with OD. A recent proof of concept study using this triple adaptation of diets in older patients with OD that were discharged from a general hospital in the context of a multimodal intervention shows an improvement in functional status and functionality, and a reduction in hospital readmissions, respiratory infections, and mortality [4]. This suggests that this triple adaptation might become a simple and cost-effective strategy in avoiding OD complications in this geriatric population.

Rheologic adaptation is critical in providing a safe and efficient swallow in older patients with OD. Multiple studies have shown a reduction in the risk of penetrations and aspirations with the modification of bolus viscosity using thickeners (evidence A and B). The health claim stating that thickening agents are a valid therapeutic strategy for oropharyngeal dysphagia and in reducing the risk of airway invasion is supported by a recent White Paper of the European Society for Swallowing Disorders (38). Thickening agents are under the category of “Food for Special Medical Purposes” and their use is regulated by a European Commission Notice on the classification of Food for Special Medical Purposes (2017/C 401/01) [56] and also under the regulation (UE) 609/2013 (37). In addition, the substance causing the health claim (type of Thickening Agent), the amount of substance (g/100 mL), and the specific viscosity level causing the therapeutic effect must also be provided on the label, according to the European regulations. The ESSD is claiming for this information (ESSD classification, EC) to be put on the label of the product, specifically: the SI units of viscosity at 50 s^−1^ and 25 °C (e.g., EC250 and EC800 for this study) and the composition, including type of thickening agent (xanthan gum in this study) [57]. This labeling system has the endorsement of 10 medical associations, including the European Federation of Associations of Dietitians and the European Geriatric Medicine Society [57]. The viscosities of 250 and 800 mPa·s were selected in our study according to our previous publications [6,26], where 250 and 800 mPa·s presented the highest therapeutic effect in patients with OD among seven different viscosities. However, α-amylase in saliva can negatively impact the therapeutic effect, decreasing the viscosity of the bolus in a relevant manner in starch-based thickeners, leading to an increasing consensus that thickeners based on xanthan gum should replace these [14]. The compliance to thickeners in general and starch thickeners in particularly is very low due to unpalatability [58], with an increased risk of dehydration. Fluid viscosity in this study for hydration and alimentary fluids was prescribed according the V-VST, a sensitive clinical method to identify patients with dysphagia and aspiration at risk of respiratory and nutritional complications, and patients whose deglutition could be improved by enhancing bolus viscosity [27].

Several food textural and rheological properties, such as elasticity, hardness, gumminess, springiness, creaminess, crispness, brittleness, chewiness, adhesiveness, and cohesiveness, are used to describe the behaviour of solid food boluses. The Japanese Society for Dysphagia Rehabilitation considers hardness (under 15,000 N/m^2^), adhesiveness (under 1000 J/m^2^), and cohesiveness (between 0.2 and 0.9) as being the most significant [59]. A future step in our investigation would be a rigorous study on these rheological properties of food in order to describe the textures of recipes with more precision. However, without this information, in our study, we defined the texture of diets based on a qualitative method, the Texture Modified Descriptors of the British Dietetic Association (BDA), which allows us to take a first step in the modification of food intake for patients with OD. After exploring the numerous terminologies for texture-modified food that is available nowadays [34,60,61], we chose the BDA as the a very well-known system in our social healthcare environment, and one with simplified levels of texture modification. Moreover, it is easy to apply in older people and is easily extrapolated to all people with OD, which is an important point for future interventions of triple-adapted diets.

Nevertheless, some limitations of the BDA classification were observed. For example, bread must be blended, but bread is very important in the Mediterranean diet, and especially in the Catalan culture where bread is rubbed with tomato and olive oil is added, and the restriction could lead to a low adherence of texture E diets as we could not include bread with tomato and oil in meal plans. In order to establish the most appropriate texture for each patient, a clinical evaluation of chewing capacity should be performed. Nowadays, many methods have been established to measure the physical properties of food that is associated with mastication and swallowing and the ability to chew solids, such as the Test of Masticating and Swallowing Solids (TOMASS) [62]. Our group is already working on a clinical bedside method to assess mastication, but until then, a clinical evaluation using videofluoroscopy or FEES, or a clinical evaluation of mastication and swallowing can be enough to prescribe the menus.

Even though texture modified solids are recommended to promote safe swallowing and reduce aspiration in patients with dysphagia, the literature on its nutritional benefit is divided [63,64]. Numerous studies have showed how texture-modified diets have difficulty in meeting patients’ requirements for energy and protein when compared with normal diets, increasing the risk of malnutrition in patients with oropharyngeal dysphagia [3,63,65,66]. One of the reasons is because the modification of the texture of food involves a dilution process and, depending on the type and amount of fluid added, the nutritional density (especially caloric and protein content) of the food can be diluted, requiring a larger portion size or volume to achieve the same level of nutrient and energy content [67]. Consequently, modified texture foods may contain lower caloric and protein density when compared to regular textures [63,67,68,69,70,71]. For this reason, a protein and energy enrichment of the texture-modified food is normally recommended [66,67]; in fact, it is this strategy that we used in our nutritional intervention. Our menus contain a higher energy and protein intake depending on the patients’ nutritional status. However, the Texture C diets are enriched to reach the same energy and protein requirements as the texture E diets.

Based on the data of a previous study that was performed by our group [2] on the nutritional status and anthropometric parameters of older patients with OD, we calculated that energy requirements were 25 kcal/kg/day for older patients with OD and normal nutritional status or risk of malnutrition, and 35 kcal/kg/day for malnourished older patients with OD. Few studies have assessed the energy needs in patients with OD. One study illustrated that patients who consumed <22 kcal/kg/day during the acute period showed significantly poorer recovery from dysphagia and poorer outcomes when compared to those who consumed >22 kcal/kg/day [72]. Nowadays, several studies suggest an aggressive nutrition with an energy intake of approximately 35 kcal/kg/day (ideal body weight) to gain weight and improve both physical and swallowing function [53,54,55]. Our energy requirements have been calculated without taking into account heterogeneous physical activity, disease status, or gender, as some studies recommend [36]. There are several reasons for this decision; (a) previous data show small differences in basal metabolism between men and women and it is not common clinical practice to modify the amounts of calories between men and women in clinical menus, (b) the populations that the study was based on were very homogenous (older patients with chronic OD), so disease status was similar in all the population and, and (c) physical activity was low in those patients [2], so we standardized a PAL of sedentary or light activity for all patients.

The amount of proteins that were calculated in our menus (1.2 g/kg/day and 1.5 g/kg/day, for the two groups of patients) agree with a large group of experts in the field of protein and aging, including the PROT-AGE study, which recommends between 1.2–1.5 g/kg/day for older people with acute or chronic illness [35,36,73]. Recommendations of up to 2.0 g/kg/day are recommended for some groups in the case of severe illness, injury, or with severe malnutrition [35,36,74,75,76]. Although OD patients can show a severe malnutrition, we established requirements of 1.5 g/kg/day instead of 2 g/kg/day, because we consider that reaching 1.5 g/kg/day is already a substantial increase, as several studies show how the protein intake in frail older people is often well below 1 g/kg/day [36,63,71,77]. In addition, we must take into account the challenge of providing 90 grams of proteins in the diet (without supplements). Some recent studies suggest different strategies for protein intake, such as spreading protein intake over all meals (consuming 25–30 g protein per meal), including high-quality protein at each meal [78], or performing physical activity [79,80]. In our standard menus, we have prioritized the adherence to menus instead of spreading protein. They reach 25–30 g of protein only in main meals but not in others to avoid important cultural and gastronomic changes (breakfast and mid-afternoon are not foods that are traditionally rich in proteins in our country), and to avoid the need for protein supplements. High-quality protein (dairy, egg, meat…) were included in each main meal. Regarding protein and exercise, recent evidence recommends a certain protein intake in combination with adequate exercise as a key concept in preventing the loss of muscle mass and function with age in sarcopenia patients [81,82,83]. Future interventions can introduce specific physical activity in addition to menus to increase protein anabolism. In order to facilitate higher levels of protein and calorie intake, we created enriched menus that contain high calorie and protein foods and an extra snack that is a home-made ONS, as recommended in the last ESPEN guidelines [36], and when the fortified menus are not sufficient, we offer commercial ONS [36]. We fully agree with the nutritional recommendations of this society and in the concept that nursing interventions, education, nutritional counseling, food modification, and oral nutritional supplements can support oral nutrition.

Few studies on the sensorial modification of food have been done on older patients and the results are unclear [84]. Changes, such as colorful meals, arranged in layers, or with spices added to try improve the appearance of the dishes, have not improved the acceptance on the part of older people in a study. Some authors attribute these unclear results to the definition of an “appealing meal appearance” is not the same for older adults, where other factors, such as transparency and familiarity, may play more important roles for this age group [85]. Moreover, in some studies the population is very old and sometimes institutionalized where sensory function could be impaired. Organoleptic modification is based not only on modifying the appearance, but also on conserving to the maximum the taste, color, and smell of the traditional dish without “artificial adornments” that can be difficult to identify by the older population. Organoleptic adaptation of OD diets is a novel and essential project to promote the pleasure of eating and ensure good nutritional status. In this study, we have made a first approximation of the tastes and preferences of the older population in Catalonia and two chefs with several Michelin Stars—Mrs Carme Ruscalleda and Mr Fermí Puig—advised us in this direction. We firmly believe that the development of recipes where older people can recognize the taste and smell of their traditional dishes when presented attractively, will have a beneficial effect on consumption, but more research is necessary in this area to better understand the preferences of older people. A previous pilot study that was performed by our group, on the so-called minimum massive intervention (MMI strategy), with a more rudimentary version of these diets and a basic intervention in patients with OD (simple nutritional management, a compensatory strategy in the management of dysphagia, and an intervention in oral hygiene) caused a significant improvement in functional and nutritional status and quality of life, and a significant decrease in general hospital readmissions for respiratory infections with increased survival after six months [4].

Some problems that that presented themselves in implementation of our menus are: changing individual gastronomic preferences, rejection of certain foods or textures (especially Texture C), or changing the number of daily meals. However, the good results with the MMI bode well for the success of our intervention. The educational tools that we have developed, such as cooking exhibitions and workshops and video-recipes for patients and carers at home, will help adherence, because there are few standardized guidelines with step-by-step recipes to acquire an optimum texture modified food, particularly purees, from a sensory and nutritional perspective [70]. Pilot cooking workshops have been an absolute success, thanks to the interest of patients and carers to receive culinary training in OD and coordination between health professionals (dietitians, nurses, speech therapists) who informed patients of their existence. For that reason, the workshops are now a part of the usual clinical practice of our hospital.

## 5. Conclusions

Developing a nutritional plan for older patients with OD based on a triple rheological, nutritional, and organoleptic adaptation and made from traditional dishes that are easily produced at home, is possible and future research will evaluate the adherence and clinical outcome of this home meal plan for patients and the effect on nutritional status, respiratory complications, hospital readmissions, and quality of life.

## Supplementary Materials

The following are available online at http://www.mdpi.com/2072-6643/11/2/425/s1, Figure S1: Main evaluations and tools necessary to prescribe the Triple Adaptation diets in older patients (>70 years) with OD based on nutritional status, severity of OD and chewing capacity, Figure S2: Recipe for noodles (fideuà) with texture E (fork mashable), Figure S3: Recipe for noodles (fideuà) with texture C (thick pure), Figure S4: Recipe for white bean salad (empedrat) with texture C (thick pure), Figure S5: Recipe for Saffron salmon with texture C (thick pure), Figure S6: Homemade Oral Nutritional Supplement rich in calories and proteins based on quark, fruit (fresh and dried) and nuts, Figure S7: Recipe for Green salad with tomato and olives with texture C (thick pure), Figure S8: Orange, Table S1: Prescription algorithm on screening visit based on nutritional status (MNA^®^-sf) and intake registry (appetite and intake), Table S2: Oral supplement prescription table for follow-up visits based on nutritional status (MNA^®^), oral intake screening and weight loss, Table S3: A week’s menu with texture C food (thick puree) for older people with OD and MNA^®^ ≥ 17 (Autumn/Winter) (*), Table S4: A week’s menu with texture C food (thick puree) for older people with OD and MNA^®^ ≥ 17 (Spring/Summer) (*), Table S5: A week’s menu with texture E food (fork mashable) for older people with OD and MNA^®^ ≥ 17 (Spring/Summer) (*),Table S6: A week’s menu with texture E food (fork mashable) for older people with OD and MNA^®^ < 17 (Autumn/Winter) (*), Table S7: Nutritional contribution of the recipe for soup with semolina adapted to two viscosities.
