# Supplementary material for: Triple Adaptation of the Mediterranean Diet: Design of A Meal Plan for Older People with Oropharyngeal Dysphagia Based on Home Cooking
Source: Nutrients. 2019 Feb 18;11(2):425. doi: 10.3390/nu11020425 (PMC6412386; doi:10.3390/nu11020425)
Supplement: Supplementary file 1 [file nutrients-11-00425-s001.pdf]

nutrients

MDPI

Concept Paper

Triple Adaptation of the Mediterranean Diet: Design of A Meal Plan for Older People with Oropharyngeal Dysphagia Based on Home Cooking

Alicia Costa* [1–3], Silvia Carrión [1,2], Marc Puig-Pey [4], Fabiola Juárez [4], Pere Clavé [1,2,5,*]

1. Gastrointestinal Physiology Laboratory, CIBERehd CSdM-UAB, Hospital de Mataró. 08304 Barcelona, Spain; acosta@csdm.cat (A.C.), scarrion@csdm.cat (S.C.)
2. Centro de Investigación Biomédica en Red de Enfermedades Hepáticas y Digestivas (CIBERehd), Instituto de Salud Carlos III. 08304 Barcelona, Spain
3. Department of Dietetics and Nutrition, Hospital de Mataró. 08304 Barcelona, Spain
4. Fundació Alicia, San Fruitós del Bages, 08272 Barcelona, Spain; marc@alicia.cat (M.P.-P.), fabiola@alicia.cat (F.J.)
5. Fundació de Recerca en Gastroenterologia. 08304 Barcelona, Spain.

* Correspondence: pere.clave@ciberehd.org; Tel. +34 93 741 77 00; Fax. +34 93 741 77 33

Supplementary Materials

Figure S1. Main evaluations and tools necessary to prescribe the Triple Adaptation diets in older patients (> 70 years) with OD based on nutritional status, severity of OD and chewing capacity.

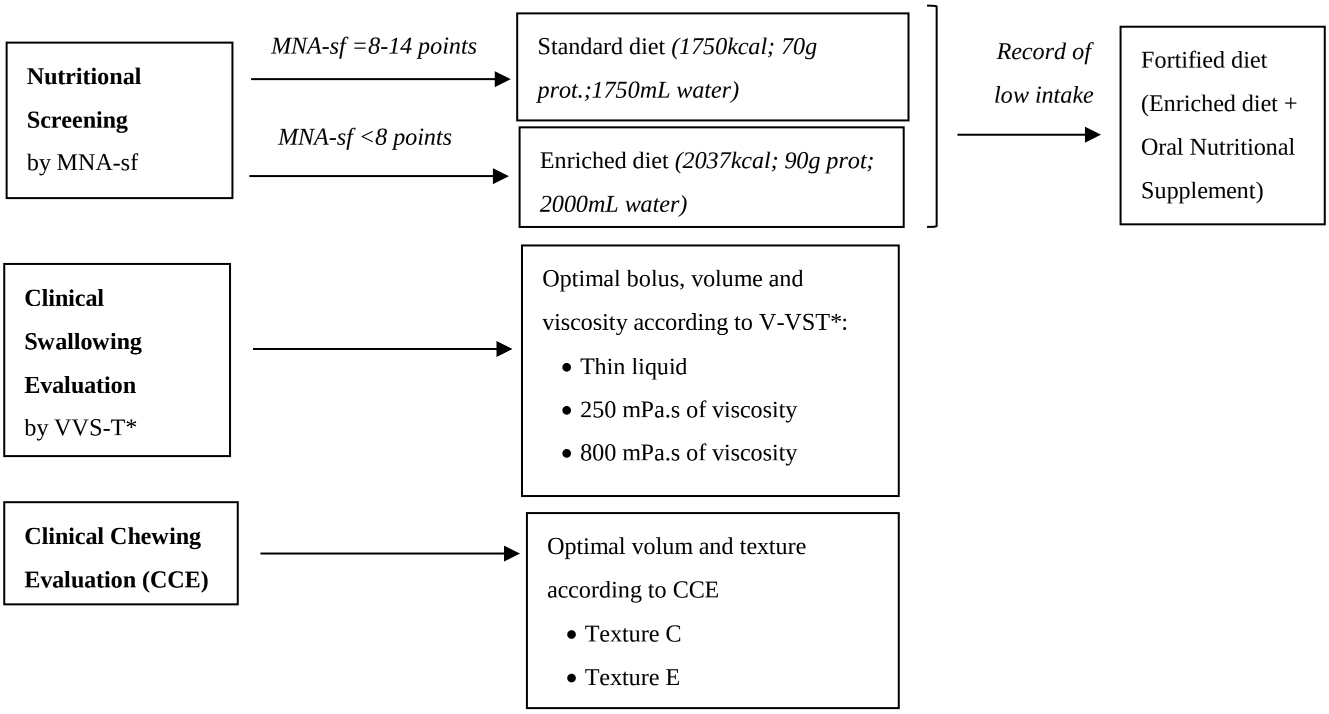

* Volum Viscosity Swallow Test

Figure S2. Recipe for noodles (fideuà) with texture E (fork mashable).

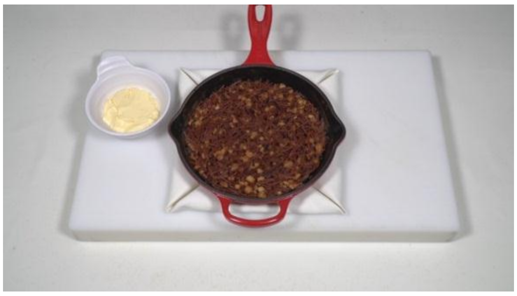

Ingredients	
Noodles nº 2	40g
Cuttlefish (clean)	30g
Shrimps, peeled	20g
Tomato, grated	20g
Onion	10g
Capsicum	10g
Garlic	½ u
Fish stock	300ml
Olive oil	15ml

Method:
1. Chop the onion and garlic very fine
2. Heat the olive oil in a pan. Fry the noodles until golden brown and put them aside.
3. In the same pan, fry the onion and garlic for 5 minutes. Add the grated tomato and the capsicum. Leave to cook for 10 more minutes.
4. Add the cuttlefish and prawns cut into small pieces. Cook for 5 more minutes and add the noodles. Stir.
5. Add the fish stock and cook until stock is completely absorbed and the pasta is well cooked and very tender, as well as the remaining ingredients.

Observations:
If the pasta is not tender enough when the stock has been absorbed, add more broth progressively until it is cooked.

Figure S3. Recipe for noodles (fideuà) with texture C (thick pure).

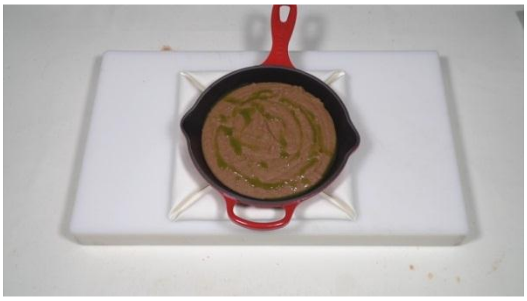

Ingredients	
Noodles nº 2	40g
Clean cuttlefish	30g
Shrimps	20g
Tomato, grated	20g
Onion	10g
Capsicum	10g
Garlic	½ u

Fish stock	300ml
Olive oil	15ml

Method:

1. Chop the onion and garlic very fine
2. Heat the olive oil in a pan. Fry the noodles until golden brown and put them aside.
3. In the same pan, fry the onion and garlic for 5 minutes. Add the grated tomato and the capsicum. Leave to cook for 10 more minutes.
4. Add the cuttlefish and prawns cut into small pieces. Cook for 5 more minutes and add the noodles. Stir.
5. Cover the noodles with the fish stock and for 7-8 minutes until the pasta is well cooked
6. Purée with the rest of the fish stock to a thick, fine and homogeneous purée

Observations:
You can accompany the noodles with a spoonful of garlic mayonnaise (allioli), mixing it into the noodles before eating. It is recommended to add the fish broth progressively while pureeing, to avoid making it too thin.

Nutritional value (per portion): Energy: 286.4 kcal; Carbohydrate: 32.68 g; Proteins: 12.89 g; Fats: 11.26g.

Figure S4. Recipe for white bean salad (empedrat) with texture C (thick pure).

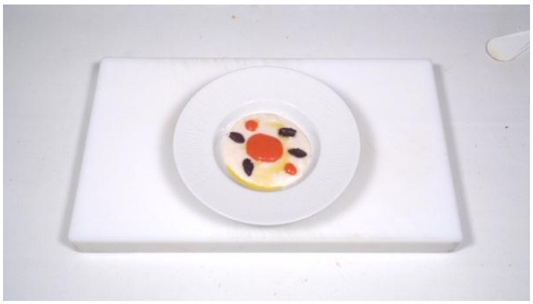

Ingredients	
White beans, boiled	100g
Onion	20g
Salt cod, soaked to remove salt	60g
Ripe tomato	60g
Capsicum	40g
Olive paste	10g
Oil	15ml
Water	50ml
Pepper	pinch

Method:
1. Cook cod in boiling water for 7 minutes. Remove from the fire
2. Mince the cod with the beans, water, and almost all the oil and pepper, forming a paste without lumps. Save.
3. Cut the red pepper, onion and tomato into pieces and mince it with a little of the oil and a pinch of pepper. Make sure there are no skins.
4. Serve the beans as a base and the minced pepper, tomato and the olive paste on top.

Observations: Stir the preparation with a spoon before eating to make sure the texture is smooth.

Nutritional value (per portion): Energy: 376.4 kcal; Carbohydrate: 22.21 g; Proteins: 27.94 g; Fats: 19.38g.

Figure S5. Recipe for *Saffron salmon* with texture C (thick pure).

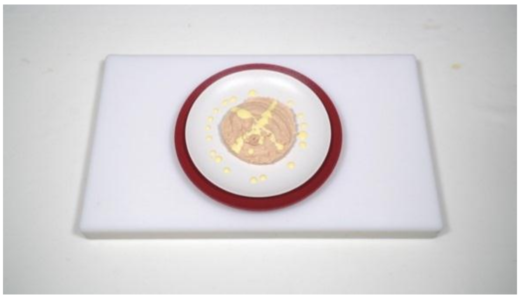

Ingredients	
For the base	
Salmon	150g
For the sauce	
Cooking cream	35ml
Saffron	40g

Method:
For the base (“papillote” salmon)
1. Heat the oven to 180ºC
2. Place the fish on a sheet of baking paper, add a little olive oil, wrap the fish in the paper.
3. Cook for 5-8 min.
4. Remove the skin.

For the sauce
1. Toast a few pieces of saffron in a hot pan.
2. Add the cream and reduce it on the fire. Remove any bits.

Observations: Serve the fish with 1 tablespoon of saffron sauce.

Nutritional value (per portion): Energy: 352.45 kcal; Carbohydrate: 4.37 g; Proteins: 31.12 g; Fats: 23.68g.

Figure S6. Homemade Oral Nutritional Supplement rich in calories and proteins based on quark, fruit (fresh and dried) and nuts.

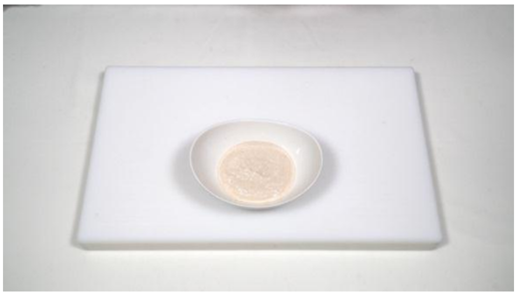

Ingredients	
Quark	150ml
Apple	130g
Ground cinnamon	to taste
Almond	20g
Raisin	20g
Sliced bread	30g

Method:
1. Soak the bread in the quark for 20 minutes for it to soften.
2. Peel and cut the apple into pieces. Cook in the microwave for 2 and a half minutes at maximum power with a little water.
3. Sprinkle with cinnamon and blend all the ingredients together until the result is a thick and homogeneous purée.

Observations: Check no small pieces of dried fruit remain. If so, strain the purée, keeping in mind that you will lose thickness and that it will be necessary to add a little more bread and blend again.

Nutritional value (per portion): Energy: 415 kcal; Carbohydrate: 63.25 g; Proteins: 18.33 g; Fats: 12.12 g.

Figure S7. Recipe for *Green salad with tomato and olives* with texture C (thick pure).

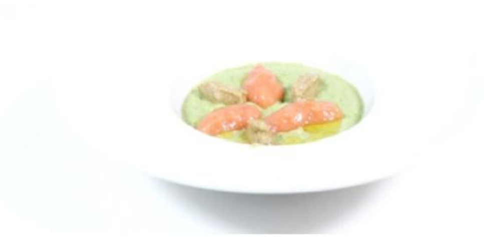

Ingredients	
Lettuce (washed)	50 g
Escarole (clean)	20 g
Lambs lettuce	10 g
Cucumber (peeled)	30 g
Onion	15g
Avocado	15g
Grated tomato	30g
Olive Paste (olivada)	5g
Virgin olive oil	5g
Vinegar	10g
Commercial Thickener	12g
Salt	pinch

Method:
1. Blend the lettuce, escarole, lambs lettuce, cucumber and onion with olive oil, vinegar and a pinch of salt. Strain.
2. Add the avocado, 6 g of a thickener and re-blend. Save.
3. Strain the grated tomato, add 6 g of thickener, remaining olive oil, a pinch of salt and blend. Save.
4. Serve the salad mixture, with spoonfuls of blended tomato simulating slices and teaspoonfuls of olive paste

Nutritional value (per portion): Energy: 102.2 kcal; Carbohydrate: 4.29 g; Proteins: 2.23 g; Fats: 8.2g.

Figure S8. Orange.

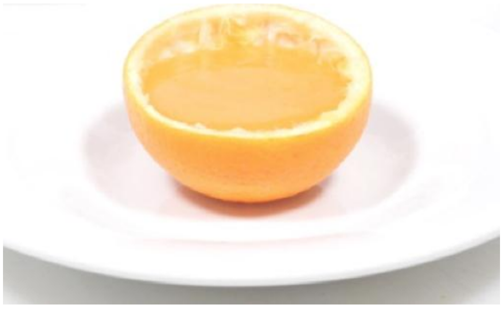

Ingredients	
Orange	1 unit
Commercial Thickener	12g

Method:

1. Cut the orange in half and make a juice. Strain
2. Add the commercial thickener and blend to get the correct texture.
3. Remove the remaining pulp from the orange halves and refill them with the thick juice.

Nutritional value (per portion): Energy: 81.6 kcal; Carbohydrate: 19.72 g; Proteins: 1.14 g; Fats: 0.32.

Table S1. Prescription algorithm on screening visit based on nutritional status (MNA®-sf) and intake registry (appetite and intake).

**Nutritional status and intake registry**	**Diet**	**Oral Nutritional Supplement**	
		**With diet (smoothie homemade)**	**With commercial product (Nutilis Complete®)**
**MNA®-sf > 11**	Standard<br>*1750 kcal*<br>*70g prot.*	----	----
**MNA®-sf 8-11**	Standard<br>*1750 kcal*<br>*70g prot.*	----	----
**MNA®-sf <8**	Enriched<br>*2037 kcal*<br>*90g prot*	Yes<br>*+460 kcal*<br>*+ 20g prot*	----
**MNA®-sf <8 and severe anorexia or very low intake**	Enriched<br>*2037 kcal*<br>*90g prot*	Yes<br>*+460 kcal*<br>*+ 20g prot*	Yes<br>≥1 u Nutilis Complete®*<br>*≥ +306 kcal*<br>+ *12g prot*

MNA®-sf: Mini Nutritional Assessment short form; ONS: Oral Nutritional Supplement
**Adjusted according to intake.*

Table S2. Oral supplement prescription table for follow-up visits based on nutritional status (MNA®), oral intake screening and weight loss.

	**Good intake (100-75%)**	**Insufficient intake** (75-50%) **or significant %WL** (5%/month)	**Very low intake** (<50%) **or severe %WL** (>5%/month)
**MNA ®> 24**	*1750 kcal 70g prot.*	*2037 kcal 90g prot*	*2037 kcal 90g prot* **2-3 u** Nutilis Complete®
**MNA® 17-24**	*1750 kcal 70g prot.*	*2037 kcal 90g prot*	*2037 kcal 90g prot* **2-3 u** Nutilis Complete®
**MNA® <17**	*2037 kcal 90g prot*	*2037 kcal 90g prot* **1-2 u Nutilis**	*2037 kcal 90g prot.* ≥ 3 u Nutilis Complete®*

MNA®: mini nutritional assessment; WL: weight loss. *Adjusted according to intake.

Table S3. A week's menu with texture C food (thick puree) for older people with OD and MNA®≥17 (Autumn /Winter) (*).

	Monday	Tuesday	Wednesday	Thursday	Friday	Saturday	Sunday
Breakfast	Milk coffee with milk and biscuits (E01)/ Orange juice (E02)	Milk coffee and cereal (E03)/ Fruit salad (E04)	Milk coffee and bicuits (E01)/Apple banana (E05)	Milk coffee and muffins (E06) / Peach juice (E07)	Milk coffee and cereals (E03) / Bread with cocoa cream (E08)	Milk coffee (E09) / Cupcakes with milk (E10)	Milk coffee with biscuits(E01) / Toast with butter and jam (E11)
kcal	*426.5*	*440.3*	*431.1*	*448*	*395.4*	*454.74*	*412.1*
prot	*9.77*	*9.24*	*9.3*	*11.22*	*9.9*	*11.27*	*9.99*
Snack	Milk coffee (*) (E09)	Peach juice (E07)	Milk coffee (*) (E09)	Milk coffee (*) (E09)	Natural yoghourt (E20)	Milk coffee (*) (E09)	Milk coffee (*) (E09)
kcal	*66.7*	*61*	*66.7*	*66.7*	*58*	*66.7*	*66.7*
prot	*1.7*	*1.07*	*1.7*	*1.7*	*3.2*	*1.7*	*1.7*
Lunch	Beans with roasted peppers and olives (D01)	Potato with tomato and anchovies (D04)	Vegetable pudding (D07)	Rice with tomato sauce and egg (D10)	Spinach with pine kernels and sultanas (D13)	Vegetables with chopped nuts (D16)	Commercial Cream (D19) (*)
	293.41	*161.62*	*242*	*333.8*	*199.4*	*136.7*	*126.24*
	14.26	*3.41*	*8.57*	*6.06*	*5.93*	*6.49*	*2.84*
	Chicken pudding (D52)	Hake in green sauce (D05)	Hamburguer with Chimichurri sauce (D08)	Mackerel with tomato sauce (D11)	Stewed chickpeas with vegetables and cod (D14)	Chicken lasagne (D17)	Paella (D20)
	309	*435.6*	*425.3*	*331.54*	*446.3*	*484.67*	*442.9*
	27.83	*29.5*	*26.3*	*26.96*	*18.29*	*23.01*	*29.25*
	Liquid yogurt (D03)	Orange (D06)	Apple (D09)	Kiwi (D12)	Fresh cheese and strawberries (E13	Flavoured yogurt (D18)	Pear (D21)
	150.5	*81.6*	*81.3*	*75.3*	*159.6*	*82*	*130.3*
	4.5	*1.14*	*0.39*	*1.15*	*8.5*	*3*	*0.77*
kcal	*752.91*	*678.82*	*748.6*	*740.64*	*805.3*	*703.37*	*699.44*
prot.	*46.59*	*34.05*	*35.26*	*34.17*	*32.72*	*32.5*	*32.86*
Dinner	Omelette with ham (S01)	Sausages with apple sauce (S02)	Courgette and potato omelette (S03)	Ham and cheese sandwich (S05)	Soup with semolina enriched (S07) (*)	Tuna sandwich (S09)	Bread with tomato and turkey spread (S11)
	432.3	*434.5*	*213.8*	*428.25*	*93.12*	*445*	*414*
	20.77	*24.3*	*15.05*	*26.36*	*6.78*	*20.65*	*21.5*
	Orange (D06)	Pear (D21)	Strawberries and oatmeal enriched smoothie (S04)	Banana (S06)	Apple. almonds and raisins smoothie (S08)	Liquid natura yogurt l (S10)	Commercial custard (S12)
	81.6	*130.3*	*282.85*	*71*	*415*	*84*	*163.8*
	1.14	*0.77*	*12.48*	*2.8*	*18.33*	*2.8*	*4.6*
kcal	*513.9*	*564.8*	*496.65*	*499.25*	*508.12*	*529*	*577.8*
prot.	*21.91*	*25.07*	*27.53*	*29.16*	*25.11*	*23.45*	*26.1*
TOTAL KCAL/ DAY	*1760.01*	*1744.92*	*1743.05*	*1754.59*	*1766.82*	*1753.81*	*1756.04*

TOTAL PROT./DAY	79.97	69.43	73.79	76.25	70.93	68.92	70.65
Kcal deviation	-10.01	5.08	6.95	-4.59	-16.82	-3.81	-6.04
Prot. Deviation	-9.97	0.57	-3.79	-6.25	-0.93	1.08	-0.65

(*) Fluids thickened to 250mPas.s

Table S4. A week's menu with texture C food (thick puree) for older people with OD and MNA®≥17 (Spring /Summer)(*).

	Monday	Tuesday	Wednesday	Thursday	Friday	Saturday	Sunday
Breakfast	Milk coffee (E15) / Bread with butter and jam (E11)	Cupcakes with milk (E10) / Apple with banana (E05)	Cereals with milk, honey and oats (E18) / Apple with banana (E05)	Cereals with milk, honey and oats (E18) / Apple with banana (E05)	Cupcakes with milk (E10) / Apple with banana (E05)	Cereals with milk, honey and oats (E18) / Apple with banana (E05)	Milk coffee (E15) / Bread with butter and jam (E11)
kcal	*397.52*	*522.6*	*399.6*	*399.6*	*522.6*	*399.6*	*397.52*
prot	*10.69*	*10.6*	*6.2*	*6.2*	*10.6*	*6.2*	*10.69*
Snack	Fruit compote (E21)	Milk coffee (*) (E15)	Fruit compote (E21)	Natural yogurt (E19)	Fresh cheese with strawberries (E13)	High protein yoghourt (Skyr) (S17)	Fruit compote (E21)
kcal	*104*	*80.52*	*104*	*70.3*	*159.6*	*97.5*	*104*
prot	*0.42*	*2.52*	*0.42*	*4.1*	*8.5*	*17.7*	*0.42*
Lunch	Aubergine and pepper pudding (D07)	Pasta salad (D36)	Chickpeas with spinach (D37)	Potato salad (D31	Green salad with tomato and olives (D47)	Rice with tomato sauce and egg (D10)	Commercial Gazpatxo (D43)
	242	*359.02*	*324.5*	*249.67*	*62.06*	*333.8*	*123*
	8.57	*6.05*	*11.9*	*3.89*	*2.22*	*6.06*	*3.11*
	White beans with cod ("Empedrat") (D34)	Cod with mushrooms (D26)	Chicken with soy and honey (D24)	Hake "a la meuniere" (D38)	Turkey with red peppers (D51)	Mackerel with tomato sauce (D11)	Hamburger with soy and honey (D32)
	376.4	*224.22*	*401.2*	*381.5*	*213.3*	*433.5*	*424.2*
	27.94	*34.11*	*38.49*	*29.3*	*25.45*	*31.29*	*37.46*
	Strawberries (D35)	Banana (S06)	Pear (D21)	Honey and cottage cheese (D57)	Fruit compote (E21)	Kiwi (D12)	Strawberries (D35)
	82.6	*76.3*	*130.3*	*140.9*	*104*	*75.3*	*82.6*
	1.15	*0.77*	*0.77*	*13.6*	*0.42*	*1.15*	*1.15*
kcal	*701*	*659.54*	*856*	*772.07*	*379.36*	*842.6*	*629.8*
prot	*37.66*	*40.93*	*51.16*	*46.79*	*28.09*	*38.5*	*41.72*
Dinner	Omelette with ham (S01)	Bread with tomato and turkey spread (S11)	Potato and onion omelette (S13)	Vegetables with herb mousseline (D60)	Tuna sandwich (S9)	Commercial vegetable cream (D55)	Pasta with carbonara (S16
	432.3	*414*	*298*	*100.5*	*445*	*126.2*	*431.52*
	20.77	*21.5*	*9.92*	*3.21*	*20.65*	*2.84*	*12.83*
	Peach with syrup (D41)	Strawberry (D35)	Banana with cherries (S24)	Banana and cocoa smoothie (S18)	Strawberry and biscuit smoothie (S20)	Strawberry and oat smoothie (S4)	Banana creme caramel (D44)
	105	*82.6*	*77.3*	*382.5*	*228.4*	*282.85*	*168.67*
	0.5	*1.15*	*0.82*	*5.54*	*8.85*	*12.48*	*4.19*
kcal	*537.3*	*496.6*	*375.3*	*483*	*673.4*	*409.05*	*600.19*
prot	*21.27*	*22.65*	*10.74*	*8.75*	*29.5*	*15.32*	*17.02*
TOTAL KCAL/ DAY	*1739.82*	*1759.26*	*1734.9*	*1724.97*	*1734.96*	*1748.75*	*1731.51*
TOTAL PROT./DAY	*70.04*	*76.7*	*68.52*	*65.84*	*76.69*	*77.72*	*69.85*
Kcal deviation	10.18	-9.26	15.1	25.03	15.04	1.25	18.49
Prot. Deviation	-0.04	-6.7	1.48	4.16	-6.69	-7.72	0.15

(*) Thickened to 800mPas.s

Table S5. A week's menu with texture E food (fork mashable) for older people with OD and MNA®≥17 (Spring/Summer) (*).

	Monday	Tuesday	Wednesday	Thursday	Friday	Saturday	Sunday

Breakfast	Milk coffee with cereals (E3) / Bread with cocoa cream (E9)	Milk coffee and biscuits (E1) / Bread with butter and jam (E13)	Milk coffee (E11) / Bread with butter and jam (E13) / Kiwi (D12)	Soy drink with Eko (E16) / Bread with cocoa cream (E9)	Milk coffee and cereals (E3) / Drinkable strawberry yogurt (E15)	Milk coffee with cereals (E3) / Bread with cocoa cream (E9)	Soy drink with Eko (E16) / Bread with cocoa cream (E9)
kcal	*395.4*	*412.1*	*373.26*	*288.7*	*405.2*	*395,4*	*288,7*
prot	*9.85*	*9.99*	*8.33*	*11.9*	*11.45*	*9,85*	*11,9*
Snack	Fruit salad (E4)	Kiwi (D12)	Strawberries (D38)	Strawberry drinkable yogurt (E15)	Apple (D9)	Natural yogurt (E10)	Peach juice (E2)
kcal	*156.22*	*75.3*	*82.6*	*121.5*	*81.3*	*58*	*122,84*
prot	*1.37*	*1.15*	*1.15*	*3.9*	*0.39*	*3,2*	*1,07*
Lunch	Aubergine and pepper pudding (D07)	Pasta salad (D36)	Pumpkin cream (D39)	Rice with tomato sauce and egg (D40)	Red lentil stew (D20)	Vegetable purée with herb mousseline (D22)	Mashed potato with mushrooms (D26)
	242	*434.82*	*231.1*	*276.8*	*205.1*	*100,45*	*195,6*
	8.57	*12.22*	*11.38*	*6.08*	*13.1*	*3,21*	*3,44*
	WhitebBeans with cod (Empedrat) (D35)	Mackerel with sauté tomato (D27)	Chicken meatballs stewed "a la catalana" (D28)	Hake with green sauce (D5)	Minced beef with demi-glace sauce (D42)	Salmon with saffron (D11)	Minced chicken with mushrooms, garlic and parsley (D21)
	376.4	*313.54*	*505.5*	*211.62*	*416*	*352.45*	*392.4*
	27.94	*30.59*	*14.7*	*27.39*	*16.25*	*31.12*	*25.28*
	Strawberries (D38)	Apple (D9)	Egg flan (D41)	Pear (D6)	Fruit salad (E4)	Fruit salad (E4)	Apple (D9)
	82.6	*81.3*	*121.5*	*130.3*	*156.22*	*156.22*	*81.3*
	1.15	*0.39*	*4.3*	*0.77*	*1.37*	*1.37*	*0.39*
kcal	*701*	*829.66*	*858.1*	*618.72*	*777.32*	*609.12*	*669.3*
prot	*37.66*	*43.2*	*30.38*	*34.24*	*30.72*	*35.7*	*29.11*
Dinner	Fish croquette (S9)	Cabbage and potato (“Trinxat”) with demi-glace sauce (D54)	Cabbage omelette (S11)	Vegetable cannelloni (S5)	Monkfish with potatoes (D48)	Stuffed aubergine (D47)	Omelette (S1)
	337.9	*275*	*287.19*	*433.4*	*258.3*	*640.33*	*518.2*
	16.4	*11.2*	*20.56*	*13.14*	*24.5*	*17.58*	*22.68*
	Drinking yogurt (E5)	Fresh cheese with strawberries (E20)	Honey and cottage cheese (D45)	Strawberry and oat smoothie (S12)	Strawberry and biscuit smoothie (S7)	Baked Pear (D29)	Drinking yogurt (E5)
	150.5	*159.6*	*140.4*	*282.8*	*228.4*	*37*	*150.5*
	4.5	*8.5*	*13.6*	*12.48*	*8.85*	*0.48*	*4.5*
kcal	*488.4*	*434.6*	*427.59*	*716.2*	*486.7*	*677.33*	*668.7*
prot	*20.9*	*19.7*	*34.16*	*25.62*	*33.35*	*18.06*	*27.18*
TOTAL KCAL/ DAY	*1741.02*	*1751.66*	*1741.55*	*1745.12*	*1750.52*	*1739.85*	*1749.54*
TOTAL PROT./DAY	*69.78*	*74.04*	*74.02*	*75.66*	*75.91*	*66.81*	*69.26*
Kcal deviation	8.98	-1.66	8.45	4.88	-0.52	10.15	0.46
Prot. Deviation	0.22	-4.04	-4.02	-5.66	-5.91	3.19	0.74

(*) Thickened to 250mPas.s

Table S6. A week's menu with texture E food (fork mashable) for older people with OD and MNA®<17 (Autumn/ Winter) (*).

	Monday	Tuesday	Wednesday	Thursday	Friday	Saturday	Sunday
Breakfast	Milk coffee (E17) + Bread with butter and jam (E13)	Milk cookies (E18) + Coca with chocolate (E19)	Cereals with milk, honey and nuts (E21) / Apple with banana (E6)	Cereals with milk, Honey and nuts (E21) / Apple with banana (E6)	Milk coffee (E17) + Toast with butter and jam (E13)	Milk coffee (E17) + Toast with butter and jam (E13)	Cupcakes with milk (E12) / Apple with banana (E6)
kcal	*397.52*	*564.5*	*399.6*	*399.6*	*397.52*	*397.52*	*522.6*
prot	*10.69*	*16.48*	*6.2*	*6.2*	*10.69*	*10.69*	*10.6*
Snack	Fresh Cheese with strawberries (E20)	Baked Pear (D29)	Commercial Fruit Compote (E22)	Commercial Fruit Compote (E22)	Fresh Strawberry Cheese (D62)	Commercial Fruit Compote (E22)	Kiwi (D12)
kcal	*159.6*	*37*	*104*	*104*	*99*	*104*	*75.3*
prot	*8.5*	*0.48*	*0.42*	*0.42*	*6*	*0.42*	*1.15*

Lunch	Vegetable puree with chopped nuts (D15)	Potato with tomato and anchovies (D4)	Red lentil stew (D20)	Cabbage and potato ("Trinxat") with demi-glace sauce (D54)	Lentils with roasted pepper and olivada (D64)	Rice with tomato sauce and egg (D40) (D40)	Vegetable puree with Romesco sauce (D7)
	136	115.28	205.1	275	259.67	276.8	59.8
	6.49	3.85	13.1	11.2	16.1	6.08	4.73
	Chicken meatballs with demi-glace sauce (D50)	Steamed hake with mushrooms (D23)	Cabbage omelette (S11)	Saffron salmon (D11)	Beef casserole with potato puree (D2)	Monkfish with potatoes (D48)	Stewed chickpeas with vegetables and cod (D14)
	416	150.9	287.19	352.45	394.7	258.3	446.3
	28.88	27.25	20.56	31.12	13.54	24.5	22.59
	Commercial custard (S12)	High protein yoghourt (Skyr) (S17)	Apple (D9)	Orange (D3)	Commercial custard (S12)	Peach with syrup (D65)	Kiwi (D12)
kcal	163.8	97.5	81.3	81.6	163.8	105	75.3
prot	4.6	17.7	0.39	1.14	4.6	0.5	1.15
Snack	Apple, almond and raisin smoothie (S08)	Apple, almond and raisin smoothie (S08)	Apple, almond and raisin smoothie (S08)	Apple, almond and raisin smoothie (S08)	Apple, almond and raisin smoothie (S08)	Apple, almond and raisin smoothie (S08)	Apple, almond and raisin smoothie (S08)
kcal	415	415	415	415	415	415	415
prot	18.33	18.33	18.33	18.33	18.33	18.33	18.33
Dinner	Omelette with potato and onion (S3)	Commercial vegeteble creme (Mediterranean Knorr) (D34)	Chicken with potatoes and rosemary (S21)	Vegetable cannelloni (S5)	Pasta with Bolognese sauce	Ham and cheese sandwich (S20)	Fish croquette (S9)
	318.87	269.4	427.9	433.4	314.5	428.25	337.9
	18.99	2.38	25.16	13.14	15.91	26.36	16.4
	Fruit salad (E4)	Bananas with cream (S30)	Red fruit smoothie (S22)	Flavoured yogurt (D56)	Flavoured yogurt (D56)	Fruit salad (E4)	Cottage cheese (S31)
	156.22	546.6	171.8	98.1	98.1	156.22	182
	1.37	3.13	7.69	3.8	3.8	1.37	10.5
kcal	475.09	816	599.7	531.5	412.6	584.47	519.9
prot	20.36	5.51	32.85	16.94	19.71	27.73	26.9
TOTAL KCAL/ DAY	2163.01	2196.18	2091.89	2159.15	2142.29	2141.09	2114.2
TOTAL PROT./DAY	97.85	89.6	91.85	85.35	88.97	88.25	85.45
Kcal desviation	-183.01	-216.18	-111.89	-179.15	-162.29	-161.09	-134.2
Prot. Deviation	-7.85	0.4	-1.85	4.65	1.03	1.75	4.55

(*) Thickened to 800mPas.s

Table S7. Nutritional contribution of the recipe for soup with semolina adapted to two viscosities.

Enriched soup with semolina (250 mPa.s)		*Enriched soup with semolina (800 mPa.s)*	
Nutritional value (per portion)		Nutritional value (per portion)	
Energy	93.12 kcal	Energy	161.7 kcal
Proteins	6.75 g	Proteins	9.27 g
Ingredients: 200mL commercial chicken broth, 20g wheat semolina, 30g egg white		Ingredients: 200mL commercial chicken broth, 40g wheat semolina, 30g egg white	
